# A polymeric piezoelectric MEMS accelerometer with high sensitivity, low noise density, and an innovative manufacturing approach

**DOI:** 10.1038/s41378-023-00628-7

**Published:** 2023-11-29

**Authors:** Chang Ge, Edmond Cretu

**Affiliations:** The Department of Electrical and Computer Engineering, The University of British Columbia, Vancouver, Canada

**Keywords:** Electrical and electronic engineering, Sensors

## Abstract

The piezoelectric coupling principle is widely used (along with capacitive coupling and piezoresistive coupling) for MEMS accelerometers. Piezoelectric MEMS accelerometers are used primarily for vibration monitoring. Polymer piezoelectric MEMS accelerometers offer the merits of heavy-metal-free structure material and simple microfabrication flow. More importantly, polymeric piezoelectric MEMS accelerometers may be the basis of novel applications, such as fully organic inertial sensing microsystems using polymer sensors and organic integrated circuits. This paper presents a novel polymer piezoelectric MEMS accelerometer design using PVDF films. A simple and rapid microfabrication flow based on laser micromachining of thin films and 3D stereolithography was developed to fabricate three samples of this design. During proof-of-concept experiments, the design achieved a sensitivity of 21.82 pC/g (equivalent open-circuit voltage sensitivity: 126.32 mV/g), a 5% flat band of 58.5 Hz, and a noise density of 6.02 µg/√Hz. Thus, this design rivals state-of-the-art PZT-based counterparts in charge sensitivity and noise density, and it surpasses the performance capabilities of several commercial MEMS accelerometers. Moreover, this design has a 10-times smaller device area and a 4-times larger flat band than previous state-of-the-art organic piezoelectric MEMS accelerometers. These experimentally validated performance metrics demonstrate the promising application potential of the polymeric piezoelectric MEMS accelerometer design presented in this article.

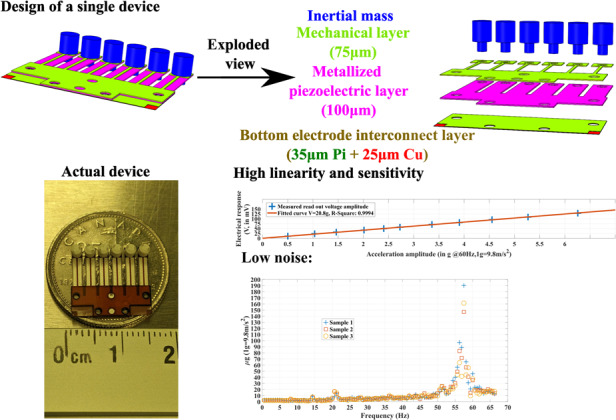

## Introduction

Microelectromechanical system (MEMS) accelerometers detect mechanical acceleration through alterations in electrical capacitance (capacitive), resistance (piezoresistive), or charge (piezoelectric) as occurs within miniaturized structures. They rank second in usage among MEMS devices, with only pressure sensors being more commonly used^1^. While traditionally used for vibration monitoring^2^, automotive testing^2,3^, and inertial navigation^3,4^, recent research highlights the potential of MEMS accelerometers in health monitoring devices and implantable hearing aids^4–7^. Early MEMS accelerometers used piezoresistive coupling in silicon. Advances in silicon micromachining enabled the robust fabrication of more intricate movable micromechanical structures, leading to capacitive accelerometers with comb drives. Piezoelectric MEMS accelerometers have therefore become popular of late. They provide many advantages over piezoresistive and capacitive couplings, such as higher temperature stability, higher robustness, reduced power consumption, better linear characteristics, wider dynamic range, enhanced sensitivity, and no vacuum sealing requirement^1–10^. Additionally, when used for implantable hearing aids, piezoelectric accelerometers have the potential to directly interface with neurons, eliminating the need for extra readout circuits^5–7^.

Despite their diverse advantages, piezoelectric MEMS accelerometers face a tradeoff of environmental exposure risks versus performance. For example, high-performance piezoelectric materials such as lead zirconate titanate (PZT) raise ecological concerns related to heavy metals^11–14^. Lead-free high-performance alternatives such as potassium sodium niobate (KNN) present sustainability-related issues during raw material excavation^11,12^. Even though more environmentally friendly materials such as aluminum nitrides (AlN)^5,7,15,16^ and zinc oxide (ZnO)^6,13,14,17^ can be used for piezoelectric MEMS transducers, they cannot provide device performance comparable with PZT-based MEMS transducers due to weaker piezoelectric properties. Moreover, all these inorganic materials, regardless of their piezoelectric properties and environmental impacts, are used in MEMS fabrication flow based on similar methodologies. Being either bottom-to-top surface micromachinings or top-to-bottom bulk micromachinings^4,8,10,13–15^, these microfabrication flows all inevitably repeat the three-step cycle of material deposition, masking lithography, and anisotropic etching, leading to almost identical process complexity. If a design opts for reduced performance for environmental benefits, then a simpler fabrication process should at least be expected to make the tradeoff worthwhile.

A viable approach to address the aforementioned challenges is the development of polymeric piezoelectric MEMS devices utilizing poly(vinyl fluoride) (PVDF) films. First, PVDF has higher piezoelectric coefficients than ZnO and AlN^18^, laying the foundation for potentially higher-performing devices. Additionally, PVDF films can be directly shaped into microstructures through advanced methods such as laser micromachining and additive manufacturing, bypassing traditional three-step cycles to streamline fabrication flows.

In addition to simultaneously achieving simple, environmentally friendly microfabrication flows that yield high sensitivity devices, polymeric piezoelectric MEMS accelerometers based on PVDF can also make a more solid foothold for MEMS inertial sensors in the emerging field of flexible electronic microsystems, giving extra significance to the research of these polymer MEMS devices. Leveraging the constant advances in semiconducting organic materials and organic field effect transistors^19^, research interest in full-polymer electronic systems featuring polymer sensors and polymer integrated circuits continues to rise, causing an increased demand for polymeric MEMS sensors as the vital front end for information collection and fueling the corresponding research. For example, tactile sensors, as the polymeric subcategory of MEMS pressure sensors, have been extensively studied, enabling diverse novel applications of this most widely used MEMS type, such as intelligent skins and soft robots^20^.

In contrast, research on developing polymer versions of MEMS inertial sensors, which are the second most-often used MEMS type, is less mature. Moreover, the few existing studies on high-performance, polymeric, piezoelectric MEMS accelerometers lean toward adapting energy harvesters as accelerometers^21–23^. MEMS energy harvesters are tuned to work around their mechanical resonant frequency, taking advantage of the corresponding most significant mechanical response. In contrast, MEMS accelerometers work on a flat band leftwards to their mechanical resonant peak. Compared to conventional MEMS accelerometers, the alternatives converted from MEMS energy harvesters have a confined bandwidth, limiting their application potential. To address this long-standing limitation, further research on polymeric conventional piezoelectric MEMS accelerometers remains necessary.

In this context, a new design for PVDF-based piezoelectric MEMS accelerometers is proposed. Three samples were fabricated using a simplified polymer-based microfabrication technology. These samples were characterized for their mechanical resonant characteristics, frequency response, flat band sensitivity to input accelerations, and device-level noises. A comparison between the experimental measurements and benchmarks indicates promising performance. Following this introduction, subsequent sections discuss the device design, test results, benchmark comparison, fabrication flow, and experimental setup. The paper concludes by summarizing the present study’s significance and future potential.

## Results

### Theoretical feasibility

The opportunity for achieving polymeric, high-performance piezoelectric MEMS accelerometers using bulk PVDF films is hypothesized based on the theoretical principles of piezoelectric sensing. The general equation for the electrical output of a piezoelectric MEMS sensor can be written as^24^:

1 $$\left(\begin{array}{l}{Q}_{piezo}=\iint [\begin{array}{ccc}{D}_{1} & {D}_{2} & {D}_{3}\end{array}]\left[\begin{array}{c}d{A}_{1}\\ d{A}_{2}\\ d{A}_{3}\end{array}\right]\\ {V}_{piezo}={Q}_{piezo}\left[\begin{array}{c}\frac{1}{{C}_{1}}\\ \frac{1}{{C}_{2}}\\ \frac{1}{{C}_{3}}\end{array}\right]\end{array}\right.$$

Equation (1) outlines piezoelectric sensing in a Cartesian coordinate system. *Q*_*piezo*_ represents the electrical charge (unit: C) produced by the direct piezoelectric effect, forming the foundation of a sensor’s sensitivity. *V*_*piezo*_ is the open-circuit voltage (unit: V) derived from *Q*_*piezo*_, which is relevant for voltage-mode piezoelectric sensors. *D*_*1*_, *D*_*2*_, and *D*_*3*_ signify the piezoelectric electrical displacements (Unit: C/m^2^). *A*_*1*_, *A*_*2*,_ and *A*_*3*_ denote the effective areas (Unit: m^2^) of the piezoelectric material. They refer to the surface area where the stress type is consistently compressive or tensile. *C*_*1*_, *C*_*2*_, and *C*_*3*_ represent the capacitors (Unit: F) in each direction. Predominantly, piezoelectric MEMS accelerometers work in the one-dimensional 3–1 mode. A piezoelectric layer is sandwiched by two electrode layers vertically, and electrical charge is induced due to the stress induced by horizontal axial elongation. Equation(1) is correspondingly simplified as^24^:

2 $$\left\{\begin{array}{c}{Q}_{piezo}=\iint {d}_{31}({\sigma }_{top}-{\sigma }_{bottom})dA\\ {V}_{piezo}=\frac{{Q}_{piezo}}{{C}_{total}}\end{array}\right.$$

In Eq. (2), *d*_*31*_ is the piezoelectric coupling coefficient (Unit: pC/N). *σ*_*top*_ and *σ*_*bottom*_ are the stresses (Unit: Pa) on the top and bottom surfaces of the piezoelectric material in response to input acceleration. Their difference, *σ*_*top*_-*σ*_*bottom*_, is pivotal for the performance of a piezoelectric accelerometer. Generally, a primary design goal for piezoelectric MEMS sensors is maximizing this value based on analysis of stress and bending in response to mechanical input. According to basic mechanical bending theories^25^, the stress exhibits the following relationship with the bending moment (Unit: N•m) and the position to the neutral axis (Unit: m):

3 $$\sigma =-\frac{M}{I}z$$

In Eq. (3), I is the moment of inertia (Unit: m^4^). Specifically, for MEMS accelerometers, the bending moment, *M*, and the moment of inertia, *I*, have the following general relationship with the effective mass (Unit: kg), *m*_*eq*_, the input acceleration (Unit: m/s^2^), *a*_*input*_, and the equivalent spring constant (Unit: N/m), *k*_*eq*_:

4 $$\left\{\begin{array}{ll}M & \propto {m}_{eq}{a}_{input}\\ {k}_{eq} & \propto I\end{array}\right.$$

Bringing Eq. (4) into Eq. (3), the stress in response to an input acceleration is linked to the mechanical resonant frequency (Unit: rad/s), *ω*, of the MEMS accelerometer as:

5 $$\left\{\begin{array}{ll}\sigma & \propto \frac{{a}_{input}}{{\omega }^{2}}\\ \omega &=\sqrt{\frac{{k}_{eq}}{{m}_{eq}}}\end{array}\right.$$

The mechanical resonant frequency, *ω*, can represent the structural characteristics of a MEMS accelerometer. Bringing Eq. (5) into Eq. (2), the general relationship between the output of a piezoelectric MEMS accelerometer, its structural characteristics, and the input acceleration is expressed as:

6 $$\left\{\begin{array}{l}{Q}_{piezo}\propto \iint \frac{{d}_{31}{a}_{input}}{{\omega }^{2}}({z}_{top}-{z}_{bottom})dA\\ {V}_{piezo}=\frac{{Q}_{piezo}}{{C}_{total}}\end{array}\right.$$

In Eq. (6), *z*_*top*_ and *z*_*bottom*_ are the distances (Unit: m) of the piezoelectric layer’s bottom and top surfaces to the neutral axis. Based on basic structure mechanics^26^, the value of *z*_*top –*_ *z*_*bottom*_ can always be approximated to the total thickness of the piezoelectric layer (Unit: m), H. Considering the general capacitance expression for a parallel-plate capacitor, the output of a piezoelectric accelerometer is linked to its structure design, piezoelectric layer thickness, and piezoelectric material properties in the following manner:

7 $$\left\{\begin{array}{l}{Q}_{piezo}\propto H\iint \frac{{d}_{31}{a}_{input}}{{\omega }^{2}}dA\\ {V}_{piezo}=\frac{{Q}_{piezo}}{{C}_{total}}\propto \frac{{H}^{2}}{\varepsilon }\iint \frac{{d}_{31}{a}_{input}}{{\omega }^{2}}dA\end{array}\right.$$

In Eq. (7), *ε* is the dielectric constant of the piezoelectric material (Unit: F/m). As per Eq. (7), under the condition that the mechanical resonant frequency of a piezoelectric MEMS accelerometer is maintained within a certain range, the sensitivity of the MEMS accelerometer can be increased by a thicker piezoelectric layer. This analysis aligns well with the conclusion of recent research on high-performance piezoelectric MEMS accelerometers based on thick PZT films (thickness over 11 µm) deposited by aerosol jet printing^4^. The latest commercial uniaxial piezoelectric PVDF films^27^ can be ten times thicker than these thick PZT films^4^. With a tailored design strategy for other influential factors shown in Eq. (7), it is possible for polymeric piezoelectric MEMS accelerometers to achieve key performance metrics rivaling their PZT-based counterparts by using thick PVDF films.

### Device design and simulation

**Fig. 1:**
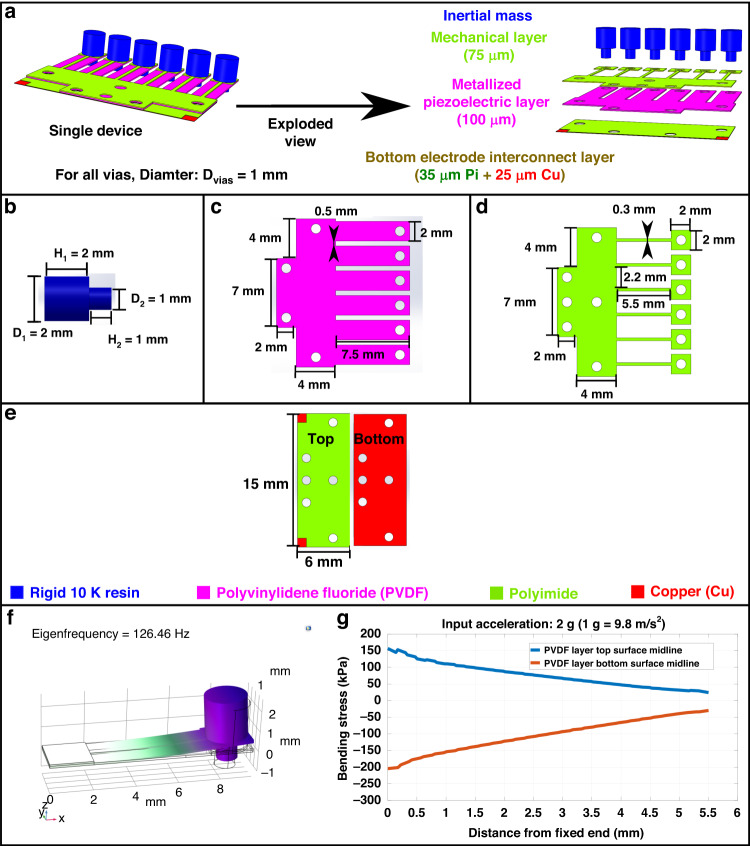
Design and simulation of the PVDF-based piezoelectric MEMS accelerometer. **a** Combined and exploded views of the device; **b**–**e** Detailed design of inertial mass, piezoelectric layer, mechanical layer, and bottom electrode interconnect layer; **f** finite element analysis for the fundamental resonant mode; **g** simulation of stress conditions on the bottom and top surfaces in response to acceleration

The present design of polymer piezoelectric MEMS accelerometers strives to achieve high sensitivity and simple microfabrication simultaneously, as detailed in Fig. 1.

As shown in Fig. 1a, every accelerometer designed in this paper has six identical cantilever-based sensing units, with the summation of their piezoelectric response as the output of the accelerometer. These six sensing units have to be electrically connected in parallel to implement this purpose, which means that the PVDF layer does not need extra patterning of its electrode layers. Cantilevers are used because they offer the unique advantage of low transverse coupling^1,4,7,16^. Using an array of sensing units can increase the reliability of a single accelerometer. More importantly, this design strategy magnifies the effect of performance optimization conducted at the level of individual sensors. The PVDF layer in Fig. 1a has a thickness of 100 µm for the optimum performance as per Eq. (7) for each sensing unit. The polyimide layer in Fig. 1a has a thickness of 75 µm to ensure acceptable structural stability.

The dimensional designs shown in Fig. 1b–d focus on achieving a low resonant frequency in each sensing unit. As per Eq. (7), a lower mechanical resonant frequency can boost the sensitivity of a piezoelectric accelerometer. The design in Fig. 1b aims to obtain a relatively large inertial mass at a miniaturized scale. In Fig. 1c, each PVDF cantilever has a length of 7.5 mm and a width of 2 mm, aiming to obtain a relatively equivalent low spring constant out of the cantilever thickness of 100 µm. For a similar reason, the width of the polyimide cantilever beam in Fig. 1d is set to 300 µm.

The polymer piezoelectric MEMS device design in Fig. 1 consists of four polymeric components that are manufactured separately and assembled into a single device. Such a device design allows the usage of direct material processing techniques for fabrication, bypassing the traditional micromachining methodology based on the three-step cycle to reduce manufacturing complexity. The vials shown in Fig. 1c to Fig. 1e are made to implement alignment for assembly and to expose the silver electrode layers. The polyimide surface in Fig. 1e is partially removed to expose the copper layer connected to the bottom electrode layer on the top surface, seeking to simplify the fabrication process. The details of the microfabrication flow are provided in a later section.

Figure 1f shows a fundamental resonant frequency of 126.46 Hz simulated in COMSOL for a single cantilever-based sensing unit. This simulation result provides a detailed scope for the resonant frequency of the design. Figure 1g shows the simulated stress conditions along the midline of the bottom and top surfaces of a single sensing unit under a 2 g (g = 9.8 m/s^2^) acceleration. The outcome details how the designed accelerometer responds to input accelerations. The stress across the top surface in Fig. 1g is consistently tensile, while the stress across the bottom surface is consistently compressive. This simulation result aligns well with the fundamental bending theories of a cantilever^25^. The minor difference in the stress magnitude indicates that the neutral axis of the composite structure is within the PVDF layer, with the top surface closer to the neutral axis than the bottom surface.

### Sample appearance

**Fig. 2:**
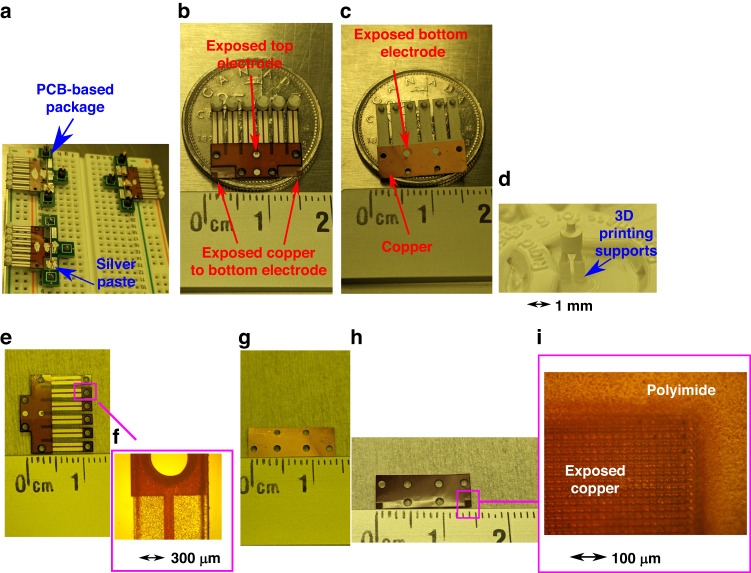
Optical images of three fabricated samples. **a** Three samples ready to test; **b**, **c** Top and bottom sides of a single sample; **d** 3D-printed inertial mass; **e**, **f** Zoomed-in view of assembled mechanical and piezoelectric layers; **g**, **h** An example of the bottom electrode interconnect layer, copper side and polyimide side; **i** Zoomed-in view of the exposed copper on the polyimide side

Three samples of the design introduced in Fig. 1 were fabricated. Figure 2 shows optical images of these samples and their components.

Figure 2a presents the appearance of three samples ready for characterization. The electrodes of the samples are accessed through customized PCB boards with the help of silver paste. Figure 2b, c highlight how the electrode layers on the PVDF film are exposed through the vias introduced in Fig. 1. Figure 2d provides insight into the 3D-printed inertial mass. Figure 2f reveals the precise alignment of the mechanical layer with the PVDF layer, demonstrating the reliability of the assembly using the vias and a common reference structure. Figure 2g, h display the copper and polyimide facets of the bottom electrode interconnect layer. The laser micromachining process left no discernible marks on the copper surface in Fig. 2g, attesting to its suitability for fabricating our accelerometer. The magnified view of the polyimide surface in Fig. 2i reveals the subtle distinctions of the exposed copper from the polyimide. The surface’s characteristic metallic sheen is visible, and minute milling traces are only visible at the microscale.

### Mechanical resonance behavior

**Fig. 3:**
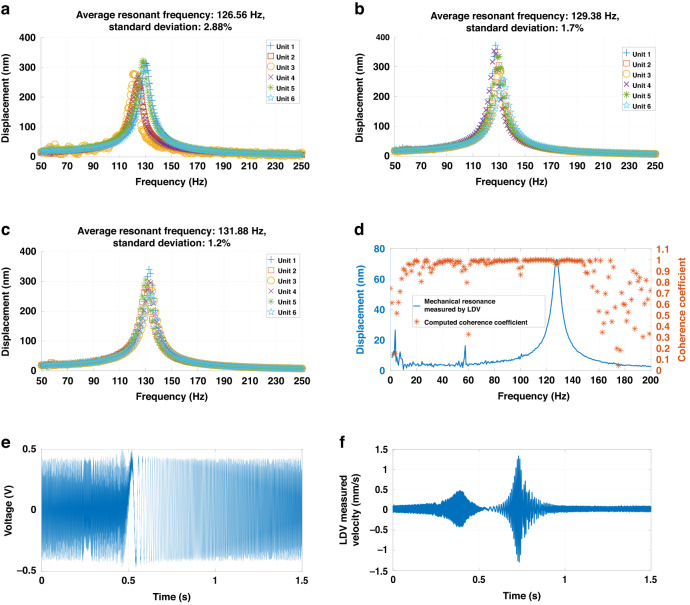
Mechanical resonant behavior of the piezoelectric MEMS accelerometers. **a**–**c** Mechanical resonance of each sensing unit for Sample 1, Sample 2, and Sample 3; **d** Coherence analysis of the LDV measurements; **e**, **f** Time-domain electrical excitation signals and measurement for the computation of coherence analysis

The three samples shown in Fig. 2 were first characterized for their mechanical resonance behaviors to extract their fundamental mechanical resonant frequency and quality factors as the base to further evaluate the performance of the three samples as accelerometers. The corresponding results are shown in Fig. 3.

All three accelerometer samples exhibited consistent mechanical resonant behaviors among their sensing elements. In Fig. 3a–c, this uniformity is represented by the low standard deviations in the resonant frequency and the high similarity in resonance profiles. The quality factors of the three samples, estimated from Fig. 3a, Fig. 3b, and Fig. 3c, are 12.5, 12.88, and 11.83, respectively.

Based on Eq. (7), the response of a piezoelectric accelerometer is affected inversely by the square of its mechanical resonant frequency. For each accelerometer sample, the consistent mechanical resonant behavior measurement indicates that all six sensing units have working interfaces for piezoelectric electromechanical coupling. Moreover, the six sensing units uniformly contribute to the total response of each sample.

Coherence analysis result shown in Fig. 3d further confirms that the most significant peaks from Fig. 3a to Fig. 3c are attributed to the samples’ fundamental resonant mode. The corresponding coherence coefficient is approximately 0.9, validating its primary origin related to the samples’ structural characteristics. This value is not precisely 1, which is attributed to the influences of ambient noise.

### Frequency response and flat band sensitivity to acceleration

**Fig. 4:**
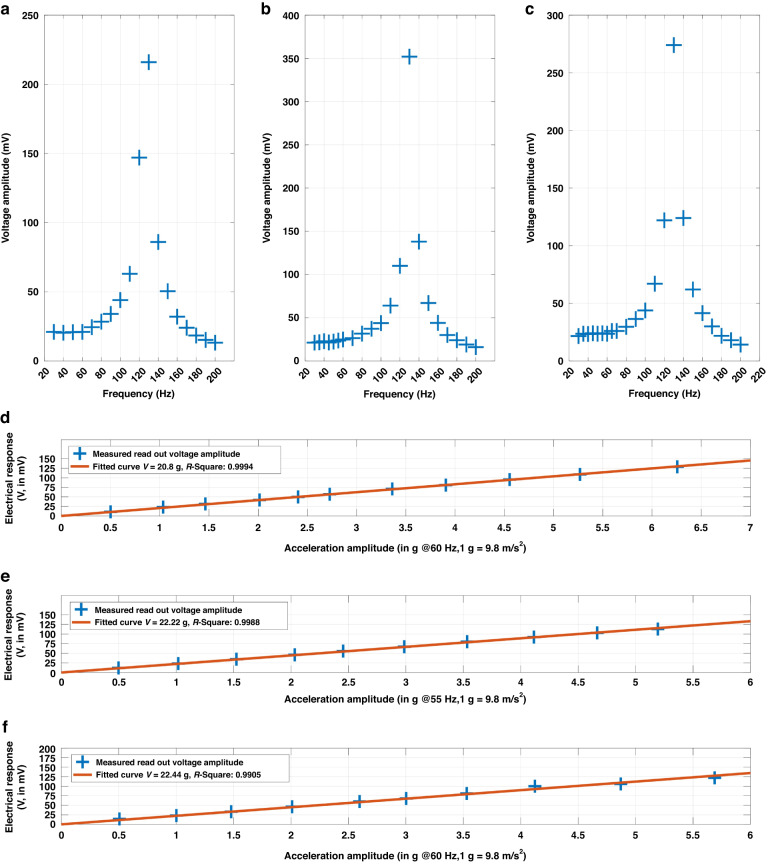
Characterization results of the three accelerometer samples. **a**–**c** Frequency response to acceleration at a magnitude of 1 g (1 g = 9.8 m/s^2^) for Sample 1, Sample 2, and Sample 3; **d**–**f** The 5% band sensitivity for Sample 1, Sample 2, and Sample 3, respectively, at the end frequency of their band

The frequency response and flat band sensitivity to accelerations are two fundamental performance metrics for an accelerometer. The detailed values of these two performance metrics provide the basis to evaluate the effectiveness of the presented design strategy. The corresponding test results are shown in Fig. 4.

Figure 4a–c present the frequency response of the three accelerometers from 20 Hz to 200 Hz under an acceleration of 1 g. The response profiles highly resemble the mechanical resonance measurement results shown in Fig. 3a–c. As the input frequency approaches each accelerometer’s resonant frequency, the measured voltage increases, reaching a peak near the mean resonant frequency before receding. These patterns confirm that the three samples can work as conventional accelerometers. Below the mechanical resonant frequency, they have a 5% flat band with a relatively constant response to the input mechanical acceleration magnitude. The edge frequencies of this 5% band are respectively 60 Hz, 55 Hz, and 60 Hz for the three accelerometers. These values were determined by a statistical method based on the cumulative average of the frequency response measurements from Fig. 3a–c. In detail, for a specific frequency value ${f}_{i}$ from $F=\{{f}_{1},{f}_{2},\mathrm{..}.,{f}_{n}\}$ and the corresponding voltage readout ${V}_{i}$ from $V=\{{V}_{1},{V}_{2},\mathrm{..}.,{V}_{n}\}$, the average voltage readout until ${f}_{i}$ is computed by:

8 $${\overline{V}}_{i}=\frac{\mathop{\sum }\limits_{1}^{i}{V}_{i}}{i}$$

The relative deviation of ${V}_{i}$ from $\overline{{V}_{i}}$ in Eq. (8) is then computed. The last ${f}_{i}$ that allows the deviation to be less than 5% is the end frequency.

Figure 4d–f shows the test results of flat band sensitivity with input accelerations from 0.5 g to 6 g. Using MATLAB for data fitting, R-square coefficients exceeded 0.99 in all three figures, suggesting that the fitted curve slope accurately represents the system-level (accelerometer and readout circuit) voltage sensitivity to input acceleration. Based on the readout circuit schematic, the system-level voltage sensitivity can be translated to the charge sensitivity. For the three samples, their charge sensitivities are 20.8 pC/g, 22.22 pC/g, and 22.44 pC/g, respectively. According to Fig. 1c, the total capacitance of a single accelerometer in this paper is approximately 172.74 pF. Based on Eq. (2), the equivalent open-circuit voltage sensitivities of the three accelerometer samples are 120.41 mV/g, 128.63 mV/g, and 129.91 mV/g.

### Device-level noise measurement and analysis

**Fig. 5:**
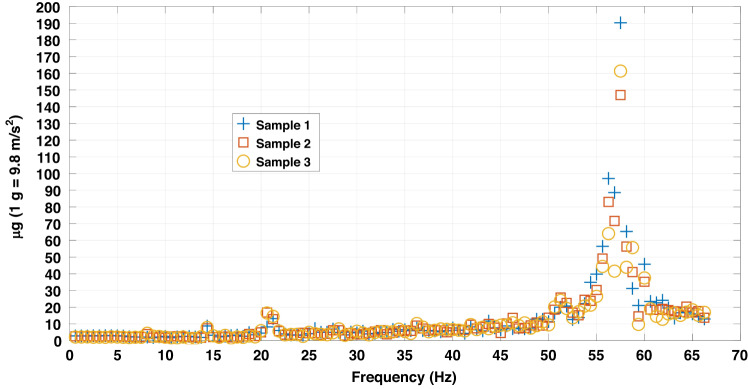
Device-level thermomechanical noise measured as acceleration for the three accelerometer samples, with the noise magnitude meausred in μg (1g = 9.8 m/s^2^, 1 μg = 10^-6^g)

The device-level noise is another crucial performance metric of a MEMS accelerometer, with lower noise signifying an ability to detect accelerations with smaller magnitudes. For the three accelerometer samples fabricated here, their thermomechanical noise profiles were measured using accelerations. These results are shown in Fig. 5.

For each accelerometer sample, the noise in Fig. 5 is the summation of the noise measured on the free ends of the six sensing units. Notable noise peaks occur at approximately 57.5 Hz across all samples. These peaks are within the 5% flat band determined in Fig. 4. However, the amplitudes of these noise peaks fluctuate between 140 and 190 µg in Fig. 5. They are three to four magnitudes smaller than the input acceleration amplitude for testing flat band sensitivity. Therefore, they do not significantly affect the measurement result in Fig. 4 or the derived performance metrics.

The coherence coefficients of the noise peaks in Fig. 5 are approximately 0.2–0.3 in Fig. 3d, ascribing their primary origin to ambient background noise rather than the inherent structure and material characteristics of the accelerometer samples. Therefore, it is reasonable to neglect measured noise between 50 and 60 Hz in Fig. 5 when estimating the accelerometers’ intrinsic noise. The thermomechanical noise and the noise density of the 5% flat band for each accelerometer are computed using the following equation:

9 $$\left\{\begin{array}{l}{a}_{m\_total}=\sqrt{{f}_{step}\mathop{\sum }\limits_{1}^{n}{p}_{i}}\\ {a}_{m}=\sqrt{\frac{{f}_{step}\mathop{\sum }\limits_{1}^{n}{p}_{i}}{{f}_{5 \% band}}}\end{array}\right.$$

The computation of Eq. (9) is based on the power spectrum density (PSD) data (Unit: m^2^/s^4^), *p*_*i*_. This value was measured by the experiment apparatus together with the noise frequency spectrum. The *f*_*step*_ in Eq. (9) is the frequency increment step used for measurement, with a value of 0.625 Hz. The *f*_*5%band*_ here is uniformly set to 50 Hz. For the three samples, the total thermomechanical noises within their 5% band are 40.63 µg, 42.61 µg, and 42.24 µg, with an average of 41.83 µg. The thermomechanical noise densities are 5.75 µg/√Hz, 6.026 µg/√Hz, and 5.97 µg/√Hz, with an average of 5.92 µg/√Hz. The standard deviation of the two parameters is 2.5%.

In addition to thermomechanical noise, piezoelectric MEMS accelerometers are also affected by thermoelectrical noise^28^. This frequency-dependent noise density can be expressed by the following equation^28^:

10 $${a}_{e}=\sqrt{\frac{4{k}_{b}T\eta {C}_{total}}{\omega {Q}^{2}}}$$

In Eq(10), *a*_*e*_ is the electrothermal noise density (unit: µg/√Hz) at a certain frequency; *k*_*b*_ is the Boltzmann constant; *T* is the temperature in Kelvin; *η* is the loss factor, which is 0.02 for PVDF^29^; *ω* is the angular frequency (unit: Rad/s) of the input acceleration; and *Q* is the charge sensitivity (unit: pC/g). Using the square of Eq.(10) as the *p*_*i*_ in Eq.(9), the thermoelectrical noise of the three accelerometer samples within their 5% band are 10.22 µg, 9.57 µg, and 9.48 µg, with an average of 9.76 µg. The corresponding noise densities are 1.45 µg/√Hz, 1.35 µg/√Hz, and 1.34 µg/√Hz, with an average of 1.38 µg/√Hz. The standard deviation of the two parameters is 4.2%. The total noise density within the 5% flat band and the corresponding noise density is computed by^28^:

11 $$\left\{\begin{array}{l}{a}_{total}=\sqrt{{a}_{m\_total}^{2}+{a}_{e\_total}^{2}}\\ {a}_{density}=\sqrt{{a}_{m}^{2}+{a}_{e}^{2}}\end{array}\right.$$

The final device-level noise within the 5% flat band, as per Eq. (11), of the three samples is 41.90 µg, 43.67 µg, and 43.29 µg, with an average of 42.95 µg and a standard deviation of 2.2%. The corresponding noise densities are 5.93 µg/√Hz, 6.17 µg/√Hz, and 6.12 µg/√Hz, with an average of 6.07 µg/√Hz and a standard deviation of 2.1%. As a common practice that is used during MEMS accelerometer characterization, the minimum detectable acceleration with over 99% certainty is typically estimated as sixfold the total flat band noise^30^. This minimum detectable acceleration is 257.70 µg for the three MEMS accelerometer samples. The noise peaks in Fig. 5 are well below this value, which further validate that the environmental noise at approximately 57.25 Hz negligibly impacts on device performances measured in Fig. 4.

## Discussion

For these three accelerometer samples, performance metrics are extracted from the experimental measurement results and listed in Table 1. Comparisons of the performance metrics with representative benchmarks are presented in Table 2.

**Table 1:** Summary and comparison of the performance metrics for the accelerometer samples

Mechanical resonance behaviors						
Average resonant frequency (Hz)			Standard deviation		Deviation to simulated resonant frequency	
128.95			2.7%		1.9%	

Performance metrics as polymeric piezoelectric MEMS accelerometer (Averaged)						
5% flat band (Hz)	Charge sensitivity (pC/g)	Open circuit voltage sensitivity (mV/g)		Noise density within the 5% flat band (µg√Hz)		Total noise of the 5% flat band (µg)
58.6	21.82	126.32		5.92		41.83

**Table 2:** Performance comparison with other accelerometers

Comparison with piezoelectric MEMS accelerometers in existing research						
Research group	Material	Device Area (mm^2^)	Resonant frequency(Hz)	flat band bandwidth (Hz)	Charge sensitivity (pC/g)	Voltage sensitivity (mV/g)
This work	PVDF	90 (15/unit)	128.95	58.6	21.82	126.32
Gong et al. ^4^	PZT	18.9	857.4	200	22.74	4.96
Lee et al.^10^	PZT	16.3	200	N/A	N/A	16.8
Hewa-Kasakarage et al.^37^	PZT	0.62	363.95	N/A	5.1	3.26
		1.02	482.3	N/A	3.43	1
Wang et al.^17^	ZnO mixed cellulose	900	84.75	16.95	22.04	N/A
Bermasconi et al.^31^	PVDF-TrFE	442	190	N/A	N/A	0.54
Debnath et al.^21^	PVDF	140	13	N/A	N/A	60.50 (@13 Hz)
Gong et al. ^32^	PVDF	450	N/A	N/A	134.59	N/A

Comparison with commercial single-axis MEMS accelerometers						
Model	Noise density (µg/√Hz)		Voltage sensitivity (mV/g)		Dynamic range (g)	Bandwidth (Hz)
This work	6.07 (5% band)		126.32		±6^*^	58.6 (5% band)
Gong et al.^4^	5.6 @20 Hz		4.96		N/A	200 (5% band)
ADXL 1001	40 @ 1 Hz		20		±100	4700 (5% band)
ADXL 1003	45 @ 100-10 kHz		10		±200	6200 (5% band)
ADXL 1005	75 @ 100–20 kHz		20		±100	9000 (5% bnad)
MS 1000 T	102 (in band)		90		±30	200 (3 dB)

^*^ This experimentally confirmed dynamic range of the three accelerometer samples corresponds to the maximum input acceleration amplitude used in the sensitivity test to obtain Fig. 4d–f

As shown in Table 1, the average resonant frequency of the three samples is 128.95 Hz, with a standard deviation of only 2.7%. The average frequency deviates from the simulated resonant frequency in Fig. 1f by 1.9%. These minor differences demonstrate that the simple fabrication method used here is predictable and reproducible.

The first part of Table 2 compares the polymeric MEMS accelerometers fabricated here to piezoelectric MEMS accelerometers reported in previous research works. The benchmark devices are based on PZT ceramics or organic piezoelectric materials such as PVDF, copolymers of PVDF, or functionalized cellulose. The three accelerometer samples fabricated here rival their state-of-the-art PZT-based counterparts^1,4^ in charge sensitivity. Moreover, the open-circuit voltage sensitivity of our polymeric piezoelectric accelerometer is over 30 times higher than that of the same PZT-based accelerometers, which, as per Eq. (7), could be caused by the large difference in the dielectric constant between PVDF ( ~ 13.5) and PZT ( ~ 1300). This comparison validates the effectiveness of our design strategy in yielding high-sensitivity polymeric piezoelectric MEMS accelerometers using PVDF films.

Notably, for the three accelerometer samples, their resonant frequency and the 5% flat band bandwidth are considerably smaller than those of the counterpart devices based on PZT. As previously discussed (with Fig. 1), our design strategy prioritizes device sensitivity over the flat band bandwidth. The lower resonant frequency and larger device area than PZT-based MEMS accelerometers in Table 2 are tradeoffs of this design strategy. Impliable from the much higher voltage sensitivities of the polymer piezoelectric MEMS accelerometers, one possible method to obtain high sensitivity and large bandwidth is using the open-circuit voltage rather than the short-circuit charge (such as the three samples in this paper) as the readout for polymeric piezoelectric MEMS accelerometers. In this respect, a major challenge will be the integration between the voltage amplifier circuit and the accelerometer to minimize the parasitic effect.

Regarding comparison with a piezoelectric MEMS accelerometer based on organic piezoelectric materials, Table 2 reveals that our polymeric MEMS accelerometers outperform these alternative devices. Our PVDF-based piezoelectric MEMS accelerometers reach a similar charge sensitivity to their counterpart based on ZnO-decorated cellulose^17^. However, the sensing unit of our accelerometers is 60 times smaller than the cellulose accelerometer in area, leading to a higher resonant frequency and a wider flat band, as shown in Table 2. This comparison result showcases that PVDF films can be used to simultaneously achieve miniaturized device design and high sensitivity for polymeric and piezoelectric MEMS transducers, which is a noteworthy advantage of PVDF over other polymeric piezoelectric materials.

In Table 2, the polymeric piezoelectric MEMS accelerometer using PVDF-TrFE has used 3D printing for micromechanical structures, with the PVDF copolymer and electrodes deposited by inkjet printing^31^. Although this additive manufacturing flow may be simpler than the fabrication flow used in this paper, the voltage sensitivity of the manufactured piezoelectric accelerometer in Table 2 is lower than the design in this paper. This comparison result indicates that, currently, direct micromachining technologies of polymer films, such as the ones used in this paper, might still be the optimum option capable of providing manufacturing robustness and high performance for polymeric piezoelectric MEMS accelerometers.

The MEMS accelerometer developed by Debnath et al.^21^ in Table 2 was converted from a PVDF-based energy harvester^21^. As outlined in the introduction, a MEMS energy harvester works around its mechanical resonant frequency, while a MEMS accelerometer works on the flat band leftwards of this frequency. For an accelerometer, the mechanical response to input acceleration is more significant around the resonant frequency than around the flat band, leading to higher sensitivity around the resonant frequency. Moreover, as per Eq. (7), the output of a piezoelectric MEMS accelerometer is inversely proportional to the square of its mechanical resonant frequency. Nevertheless, for a PVDF accelerometer that is converted from an energy harvester, despite a ten-times smaller resonant frequency than our piezoelectric accelerometers, its peak voltage sensitivity (around its mechanical resonant frequency of 13 Hz) in Table 2 remains two times lower than the flat band sensitivity of the three accelerometer samples in this paper. Notably, the PVDF film used for this energy harvester has a thickness of only 28 µm^21^, while the design of conventional MEMS accelerometers here included a thickness of 100 µm. Therefore, the higher voltage sensitivity corresponding to the design in this paper validates the performance-boosting effect of thicker piezoelectric layers in Eq. (7). More importantly, this comparison demonstrates the possibility of developing polymeric piezoelectric conventional MEMS accelerometers using PVDF films, addressing the challenges in this field that are outlined in the introduction.

The PVDF-based piezoelectric accelerometer developed by Gong et al.^32^ also used cantilever-based structures. One highlight of their achievement is the 6-times higher charge sensitivity than the accelerometers presented in this article. As shown in Table 2, the sensing area of their device is 5 times larger than that of the MEMS accelerometer presented in this work. In addition, their PVDF film is twice as thick (200 µm)^32^. As per Eq. (7), the electrical charge response is positively related to the sensing unit area and proportional to the piezoelectric layer thickness, providing a possible explanation for the higher sensitivity.

The second part of Table 2 compares piezoelectric MEMS accelerometers developed in previous research with commercially available capacitive MEMS accelerometers, with a focus on noise level and voltage sensitivity. The polymeric piezoelectric accelerometer developed in this work and a state-of-the-art PZT-based piezoelectric accelerometer show lower noise density than their capacitive counterparts, which is attributed to the inherent advantages of the piezoelectric coupling principle^1,2,4,8^. The noise density of the polymeric piezoelectric accelerometers developed in this paper is slightly larger than that of their PZT-based counterparts^4^. The dominating noise source for the polymer accelerometers is the thermomechanical noise, which is over 4 times larger than the thermoelectrical noise. For the high-performance PZT piezoelectric MEMS accelerometer, its dominating noise source is the thermoelectrical noise, which is over two orders of magnitude larger than the thermomechanical noise^4^. This difference can be attributed to the material properties. Generally, polymers have more material-related viscoelastic damping than metals and ceramics. This conclusion is well reflected by the much lower quality factors of the three accelerometer samples in Fig. 3 than their PZT-based counterparts^4^, explaining the more significant thermomechanical noise in the polymeric accelerometers. Moreover, PZT has a 100 times higher dielectric constant than PVDF^4,27^, leading to more significant thermoelectrical noise, as per Eq. (10). As shown in Table 2, the tradeoff for the lower noise in piezoelectric MEMS accelerometers is a reduced bandwidth. This comparison result indicates that piezoelectric MEMS accelerometers can be used as high-performance alternatives to capacitive accelerometers in applications such as low-frequency vibration/noise monitoring. In this respect, the application potential of polymeric piezoelectric MEMS accelerometers is further enhanced by their high voltage sensitivity, low device-level noise, simple microfabrication flow, and more environmentally friendly raw materials without heavy metals.

## Material and methods

### Microfabrication flow

The materials used to fabricate the three accelerometer samples are summarized in Table 3. The detailed fabrication results have been previously presented in Fig. 2.

**Table 3:** Material, equipment, process, and recipe used in the microfabrication flow

Process: Spin-coating of adhesives											
Equipment		Laurell^®^ WS-650-23B									
Material		Manufacturer				Speed (RPM)	Duration (s)		Thickness (µm)	Baking temperature (°C)	Baking duration (s)
QPAC-40 Polypropylene Carbonate (PPC)/Acetone solution (25 wt%)		Empower Materials, USA				2500	60		<1	80	600

Process: Laser micromachining											
Equipment			Oxford Lasers^®^ Laser micromachining system								
Material			Manufacturer			Relative intensity		Laser moving speed (mm/s)		Repeat times	
Piezoelectric PVDF^*^			Poly-K USA			85%		1		7	
Kapton^®^ Polyimide			DuPont USA			100%		0.5		6	
Pyralux^®^ AC Cu-Pi composite			DuPont USA			100%^*^		3.5^**^		6^**^	
						100%^**^		0.5^***^		8^***^	

Process: Stereolithography 3D printing											
Equipment			Formlabs^®^ 3 L SLA 3D printer								
Material			Manufacturer			Parts printed			Purpose		
Rigid 10 K Resin			Formlabs USA			Inertial mass			Accelerometer component		
						Assembly case A			Reference for alignment		
						Assembly case B			Protective case cover		

Process: Roll-to-roll lamination for assembly											
Equipment	Speed (mm/s)				Temperature (°C)						
Fortex Engineering^®^ Model 304	2			60							

^*^ The manufacturer of the PVDF thin film already poled it by a corona discharge process and screen-printed 2-µm thick silver ink on both surfaces

^**^ This process involves milling to expose the copper layer

^***^ This process is to shape the electrical interconnect layer

In Table 3, laser micromachining of off-the-shelf thin films and stereolithography 3D printing of glass-fiber enhanced resin are used as the direct micromachining techniques to implement the fabrication strategy introduced in Fig. 1. The assembly of the manufactured components uses an adhesive lamination based on PPC. Specifically, before laser micromachining to manufacture the mechanical layer in Fig. 1, PPC was deposited on both the bottom and top surfaces of the polyimide thin film. The softbaking after spin-coating in Table 3 aims to solidify the deposited PPC film on one surface before deposition on the other. The solidified adhesives were melted during the heated lamination to ensure the firm combination of each component. The temperature used for the heated lamination can melt the adhesives without reducing the piezoelectric properties of the PVDF film.

Unlike conventional micromachining techniques, the microfabrication flow detailed in Table 3 skipped the repetition of material deposition, masking lithography, and selective etching. Moreover, the fabrication of the four components for a single accelerometer does not have to follow a specific sequence. This microfabrication flow is a typical example showcasing the advantages of polymers related to simpler and more flexible MEMS microfabrication.

### Experiment setup

**Fig. 6:**
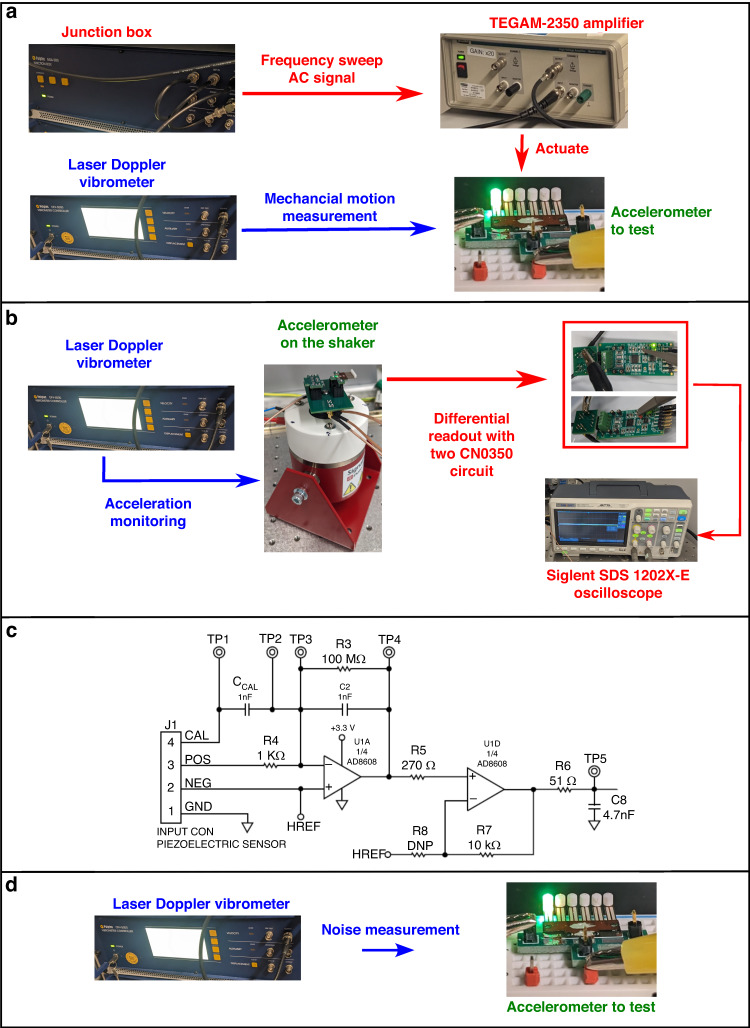
Experimental setup to characterize the polymeric MEMS accelerometers based on PVDF. **a** For mechanical resonant behavior measurement; **b** For acceleration frequency response and flat band sensitivity test; **c** Circuit schematic of the CN0350 board as readout circuit; **d** For thermomechanical noise measurement setup

The experimental setups to characterize the three polymeric piezoelectric MEMS accelerometer samples are depicted in Fig. 6, using photos of the actual apparatuses and devices.

The core apparatus used in Fig. 6 is the Polytec^®^ MSA-500 laser Doppler vibrometer (LDV) system. The mechanical resonance measurement in Fig. 6a is the most typical application of this system^33^. The mechanical resonance measurements in Fig. 3a to Fig. 3c used an AC periodic chirp signal with an amplitude of 2 V internally within the LDV system for the frequency sweep. The same type of excitation signal was used for the measurement in Fig. 3d–f, with an amplitude of 0.5 V. Area scanning with an array of measurement points covering the cantilever surface was conducted. The average value of the measurement results at all points on each cantilever is used to show the mechanical resonance behavior in Fig. 3.

The setup in Fig. 6b was used for the measurement results shown in Fig. 4. The LDV system was used to monitor the amplitude of the input mechanical accelerations applied by a Dataphysics^®^ V4 shaker. Existing research has used the Polytec^®^ LDV system in this way for the sensitivity test of a polymeric strain-gauge MEMS accelerometer^34^, supporting the suitability of the setup in Fig. 6b. Two Analog Devices^®^ CN0350 circuit boards were used for differential measurement of the output of the polymer piezoelectric MEMS accelerometers developed in this paper as a way to minimize the impact of background electromagnetic noise. Figure 6c shows the circuit board schematic. The readout voltage at TP5 is:

12 $${V}_{out}={V}_{REF}+\frac{{Q}_{piezo}}{{C}_{2}}$$

Equation (12) correlates the piezoelectric charge in response to input accelerations with the readout voltage. In Fig. 6d, the thermomechanical noise inherent to the polymeric piezoelectric MEMS accelerometers developed in this paper was measured by the LDV system without excitation. The suitability of using the LDV system for this purpose is supported by existing research works for MEMS noise characterizations^35,36^.

## Conclusion

This paper presents a new design of polymeric piezoelectric MEMS accelerometers. To the best of the authors’ knowledge, this is the first polymeric conventional piezoelectric MEMS accelerometer design based on PVDF. Laser micromachining and 3D printing were used to fabricate three samples for experimental proof-of-concept. These samples achieved a charge sensitivity of 21.82 pC/g (equivalent open-circuit voltage sensitivity: 126.32 mV/g), a 5% flat band of 58.5 Hz, and a noise density of 6.02 µg/√Hz. The designs presented in this paper rival state-of-the-art piezoelectric MEMS accelerometers based on ultrathick PZT films in sensitivity and noise density, surpassing several commercial capacitive MEMS accelerometers. Compared with the state-of-the-art organic MEMS accelerometers, the flat band of our PVDF accelerometer is four times larger. Moreover, our device is ten times smaller. In addition to the competitive performance, our polymer MEMS design has a simple, flexible, reproducible, and predictable microfabrication flow not using piezoelectric materials with heavy metals. These results support that our design can be used as a more environmentally friendly alternative to traditional high-performance PZT-based piezoelectric MEMS accelerometers, demonstrating its potential in many applications. More importantly, the proof-of-concept work provided in this paper demonstrates that high-performance conventional piezoelectric MEMS accelerometers can be directly implemented using PVDF thin films rather than converting a PVDF energy harvester into an accelerometer as an indirect solution. Considering that MEMS accelerometers are the second largest category of MEMS products after pressure sensors, the work in this paper fills a long-missing gap in the field of polymer MEMS, paving the way for future research, such as full-polymer inertial sensing systems with polymeric MEMS accelerometers and organic integrated circuits.
